# Muscle-Bound Primordial Stem Cells Give Rise to Myofiber-Associated Myogenic and Non-Myogenic Progenitors

**DOI:** 10.1371/journal.pone.0025605

**Published:** 2011-10-14

**Authors:** Elad Segev, Gabi Shefer, Rivka Adar, Noa Chapal-Ilani, Shalev Itzkovitz, Inna Horovitz, Yitzhak Reizel, Dafna Benayahu, Ehud Shapiro

**Affiliations:** 1 Department of Computer Science and Applied Mathematics, Weizmann Institute of Science, Rehovot, Israel; 2 Department of Cell and Developmental Biology, Sackler School of Medicine, Tel-Aviv University, Tel-Aviv, Israel; 3 Department of Biological Chemistry, Weizmann Institute of Science, Rehovot, Israel; 4 Department of Biological Regulation, Weizmann Institute of Science, Rehovot, Israel; Centro Cardiologico Monzino, Italy

## Abstract

Myofiber cultures give rise to myogenic as well as to non-myogenic cells. Whether these myofiber-associated non-myogenic cells develop from resident stem cells that possess mesenchymal plasticity or from other stem cells such as mesenchymal stem cells (MSCs) remain unsolved. To address this question, we applied a method for reconstructing cell lineage trees from somatic mutations to MSCs and myogenic and non-myogenic cells from individual myofibers that were cultured at clonal density.

Our analyses show that (i) in addition to myogenic progenitors, myofibers also harbor non-myogenic progenitors of a distinct, yet close, lineage; (ii) myofiber-associated non-myogenic and myogenic cells share the same muscle-bound primordial stem cells of a lineage distinct from bone marrow MSCs; (iii) these muscle-bound primordial stem-cells first part to individual muscles and then differentiate into myogenic and non-myogenic stem cells.

## Introduction

Skeletal muscles and axial skeleton share the same embryonic origin, the mesoderm [1], [2], [3], [4]. Skeletal muscles of the limb and trunk and their resident stem cells, namely satellite cells, arise from mesodermal somites, whereas the origin of head muscles and their satellite cells is from the non-segmented mesoderm [5], [6], [7], [8]. The embryonic origin of mesenchymal stem cells (MSCs) may be from other regions of the somitic mesoderm [9], [10], [11].

In the adult, MSCs were first identified as a stromal population (distinct from hematopoietic stem cells) in the bone marrow and were then identified in virtually all adult organs. MSCs were shown to be capable of forming bone, cartilage, adipose, and to a much lesser extent muscle [12], [13], [14].

In the adult, satellite cells contribute myogenic progeny that account for postnatal growth, maintenance and regeneration of skeletal muscles [15], [16]. Satellite cells reside between the basement membrane and the sarcolemma of individual muscle fibers (myofiber). Myofiber cultures give rise to myoblasts but also to non-myogenic cells such as adipocytes or fibroblasts [17], [18], [19], [20], [21], [22], [23]. The nuclei of myofibers do not possess the ability to proliferate (i.e., are post-mitotic), therefore the origin of non-myogenic cells in cultured myofiber could be either satellite cells or cells that adhered to the myofiber surface. The notion that satellite cells maintain mesenchymal differentiation plasticity is conceivable since mesenchymal and myogenic progenitors arise from the embryonic mesoderm [24], [25]. Moreover, we previously showed that non-myogenic clones are composed of fibroblasts and/or adipocytes similar to the composition of MSC progeny [20]. Alternatively, non-myogenic cells identified in primary myogenic cultures may be the progeny of non-satellite stem cells, such as MSCs, that have been co-isolated with myofibers [26]. Indeed, it was previously suggested that MSCs from the muscle interstitium account for non-myogenic cells that form in the muscle tissue [27], [28], [29]. Part of these MSCs may originate from the bone-marrow that is recognized to contain circulating MSC cells [30].

To date the lineal relations between myofiber-associated myogenic (MA-M) and non-myogenic (MA-NM) progenitors is unknown, and addressing this question is the main aim of this study. To achieve this, we opted to determine the lineage relationships between myogenic and non-myogenic progenitors from several muscles of different embryonic origins (i.e., the right and left Gastrocnemius limb, somite mesoderm) and the Masseter (mastication, non-somite mesoderm). These clones were compared to bone marrow derived MSCs.

We applied a method of cell lineage tree reconstruction developed in our laboratory [31], [32], [33], [34], [35]. This method, which was also applied by others [36], [37], [38], [39], is based on the fact that somatic mutations that accumulate during normal cell division endow each cell of the body with a unique genomic signature [32]. The cellular genomic signature used in the current study is derived from a set of microsatellite (MS) loci in mismatch-repair (MMR) deficient mice (Mlh1−/−). The MS mutation rate of these mice is much higher than that of wild type mice, thus increasing the accuracy of the cell lineage analysis. These mice exhibit normal morphology, but are infertile and develop cancer spontaneously [40]. Most recently, we demonstrated the reliability of this method for the detection of stem cells dynamics in the colon [41].

Myofibers and bone marrow were extracted and cells were seeded at clonal density. The DNA from these clones was amplified and served the basis for reconstruction of the lineage trees based on the fact that the genome of a clone reflects, on average, the genome of its founder [32].

It is noteworthy that cell lineage reconstruction can be applied to cells or clones from the same organism only. Cells taken from different organisms are generally incommensurable and would give rise to distinct cell lineage trees, the root of each would be the presumed zygote of that organism. Nevertheless, cell lineage trees of different organisms may have common features, e.g. similar topology and similar depth (number of cell divisions since the zygote) for cells of the same type or with the same biological markers. In addition, cell lineage trees may share common trends affected by age or disease progression, e.g. increase in the depth of certain cell types. Cell lineage analysis can and should be repeated to different organisms in order to explore such common features or common trends.

**Figure 1:**
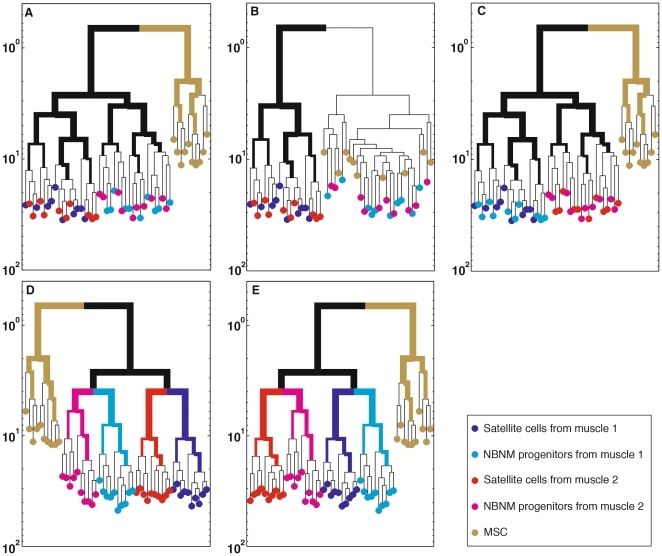
Lineage trees representing five hypothetical scenarios regarding the developmental paths of MSCs, myofiber-associated myogenic and non-myogenic progenitors. These hypothetical scenarios are composed of the following types of cells: Myofiber-associated cells extracted from a specific muscle termed here as Muscle 1 (full blue circles represent myogenic progenitors, full light blue circles represent non-myogenic progenitors); myofiber-associated cells extracted from a different muscle, termed here Muscle 2 (full red circles represent myogenic and full pink circles represent non-myogenic progenitors); and MSCs (represented by full brown circles). In scenario (A) are depicted three independent stem cell lineages: MA-M cells, MA-NM stem cells, and MSCs; In scenario (B) MA-NM progenitors and MSCs belong to the same cell population, which has a developmental path distinct from MA-M cells [27], [28]; In scenario (C) satellite cells retain mesenchymal plasticity and may stochastically become myogenic or non-myogenic [20]. The last two scenarios postulate the existence of muscle-bound stem cells of a lineage distinct from MSCs that give rise to both myogenic and non-myogenic stem cells; In scenario (D) muscle-bound stem cells first differentiate into myogenic and non-myogenic stem cells, both of which migrate independently to the individual muscles and in scenario (E) muscle-bound stem cell first migrate to individual muscles and then differentiate into myogenic and non-myogenic stem cells within each muscle. Our work attempts to resolve which of these five scenarios hold.

Several scenarios can be hypothesized regarding the lineal relations between MA-M, MA-NM and bone marrow MSCs, each resulting in a different type of cell lineage tree, as depicted in Figure 1:

- There are three independent stem cell lineages: mesenchymal, MA-M and MA-NM (Figure 1A).
- MA-NM progenitors belong to and are indistinguishable from the MSC lineage (Figure 1B) and are of a lineage distinct from MA-M.
- Satellite cells have a bi-potent potential, retaining mesenchymal plasticity, and may stochastically become myogenic or non-myogenic (Figure 1C).
- Primordial stem cells first differentiate into myogenic and non-myogenic stem cells, which migrate independently to individual muscles (Figure 1D).
- Primordial muscle-bound stem cells first migrate to individual muscles and then differentiate into myogenic and non-myogenic stem cells within each muscle (Figure 1E).

Our data show that (i) The lineage of myofiber-derived non-myogenic clones is significantly closer to that of myogenic clones than to the lineage of bone marrow MSCs; (ii) Myofiber-associated myogenic and non-myogenic progenitors have common ancestors, which we term primordial stem cells, of a lineage different from bone marrow MSCs; (iii) muscle-bound primordial stem cells first part into the different muscles and then differentiate into myogenic and non-myogenic progenitors.

## Results

Two mlh1−/− mice, M1 (330 days-old) and M2 (44 days-old) served for reconstructing the lineage trees. From these mice we extracted MSCs from the bone marrow, myofiber associated cells were extracted from individual myofibers from the left and right Gast and right Masseter aiming to elucidate the lineage relationships between cells that are tightly associated with an individual myofiber. Each individual myofiber contains, in addition to a few hundreds of post-mitotic nuclei, a few tens of satellite cells (depending on muscle type and age) and possibly some adherent cells adding to a total of much less than 100 cells per myofiber. Here we were interested in deciphering the lineal relationships between myogenic and non-myogenic ancestor cells that were derived from the same myofiber. Using a FACS method for isolating quiescent satellite cells was not applicable in this study due to the small amount of cells per myofiber. Even if the founders of the non-myogenic clones are adherent cells the total number of cells per myofiber (i.e., adherent cells and an average of 24 satellite cells per a Gast myofiber) would have been several tens, a number that is far from the minimum number of cells needed for FACS analysis. In view of this limitation, cells were separated from their parent myofiber using enzymatic and physical means and were then cultured at clonal density (see Material and Methods). These released myofiber associated cells gave rise to MA-M and MA-NM clones. The analysis of M1 is detailed bellow; the analysis of M2 revealed similar results and is detailed in the supplemented materials.

### Myofiber-associated and mesenchymal stem cells have different origin

**Figure 2:**
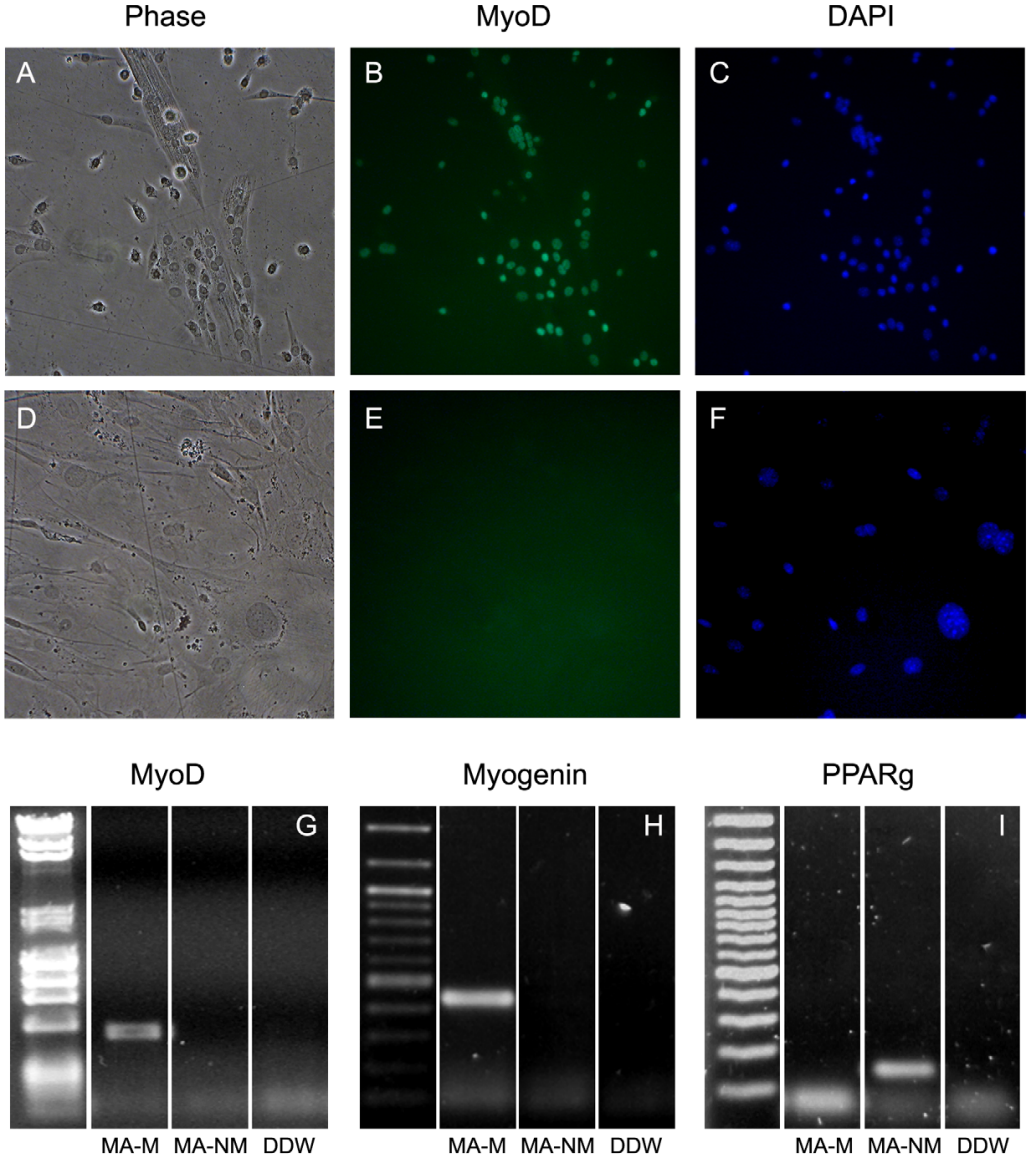
Cell morphology and mRNA expression of myogenic and adipogenic associated transcription factors, in MA-M (A–C, G–I) and MA-NM (D–F, G–I) clones derived from a Gast myofiber. (B,E) Fluorescent images of 14-day-old clones labeled with anti-Myo-D (green) and nuclei visualized with DAPI (C,F; blue). The myogenic clone (A–C) is composed of spindle-like cells and myotubes, all nuclei are labeled with MyoD. The non-myogenic clone (D–F) is composed of fibroblast-like cells and none of the nuclei is labeled with MyoD. PCR reactions were performed using cDNA prepared MA-M and MA-NM clones (G–I), the latter clone contained cells with an adipogenic phenotype. MyoD and myogenin are skeletal muscle specific transcription factors and PPARγ is a key regulator of adipogenesis. mRNA of MyoD and myogenin were expressed only in MA-M clone and PPARγ was expressed only in the non-myogenic clone.

**Figure 3:**
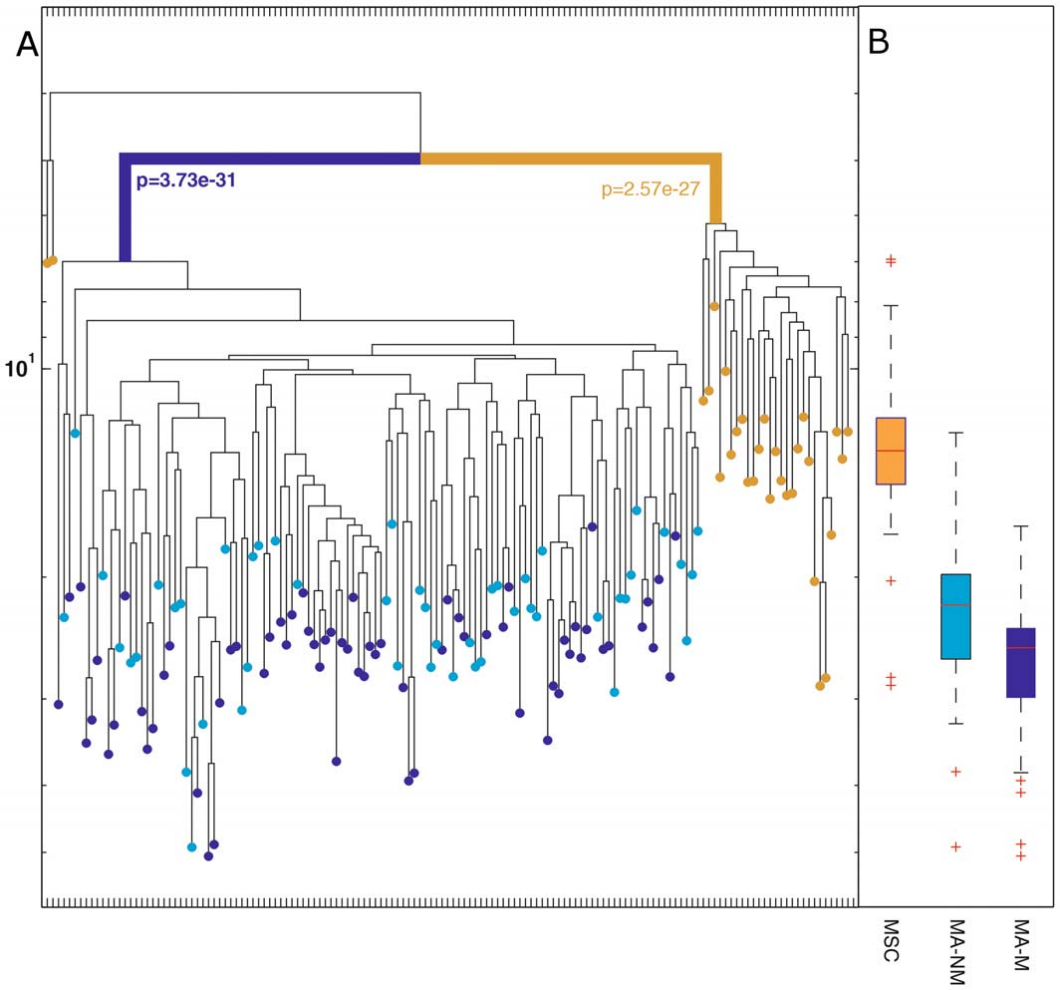
(A) Lineage tree of 116 myofiber-associated cells (68 myogenic in blue, and 48 non-myogenic in light blue), and 29 MSC (brown) from a 330 day old mouse. Each terminal node (•) represents the ancestor of a single sampled clone. The vertical axis represents cell depth. Blue and brown lines indicate significant clustering of cells. The clustering of myofiber-associated cells is significantly different than that of MSCs (p<1e-26); (B) Boxplot of the depth of myofiber-associated and MCS cells extracted from a 330 days old mouse. The box represents the spread of the middle 50% data regarding the depth of all tested clones and the red lines represent the median value of depth. Whiskers at the ends of vertical lines indicate the minimum and maximum depth values. The range in the 25th to 75th percentiles of all data was 11.8–14.6 for MSCs, 19.9–26.2 and 23.7–29.7 for myofiber-associated, non-myogenic and myogenic cells, respectively.

We isolated 116 myofiber-associated progenitors and 29 MSCs from M1. Myofiber- associated progenitors were extracted from the Gast and Masseter muscles and cloned. These cells gave rise to both myogenic (MA-M) and non-myogenic (MA-NM) clones and were characterized as shown in Figure 2. MSCs were isolated from the bone marrow of the femur and tibia, and were cultured at clonal density. Clones were harvested, DNA was extracted and subjected to mutation analyses. The cell lineage tree that was reconstructed based on these mutations shows that (i) the founders of myogenic and non-myogenic clones were clustered separately from MSCs and (ii) these two cell populations have different depths, where MSCs undergo about half the divisions that myofiber-associated cells do, median depth of MSCs is 13.2 and 24.8 for myofiber-associated cells (Figure 3). We note that some of the analyzed non-myogenic clones contained cells with an adipogenic phenotype (cells with large droplets of presumably triglycerides) and these clones clustered together with the rest of the non-myogenic clones. (Similar results from M2 are presented in Figure S2).

These data suggest that there is a common ancestor for the founders of the myogenic and non-myogenic clones in the same muscle, termed muscle-bound stem cells. These ancestors are distinct from the ancestors of bone marrow MSCs, postulating that bone marrow MSCs are not the founders of the developing non-myogenic clones.

### Muscle-bound stem cells first part into the different muscles and then differentiate into myogenic and non-myogenic stem cells

**Figure 4:**
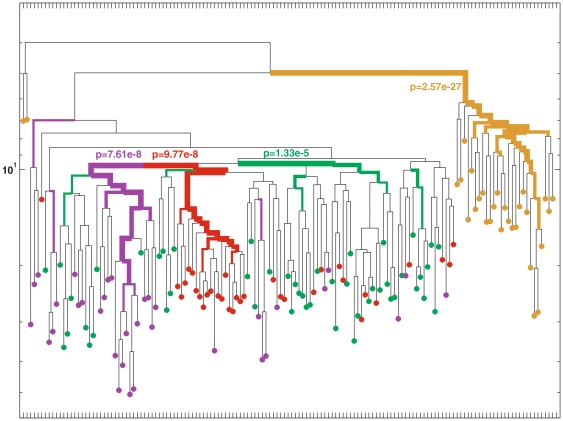
Clustering of MSCs and myofiber-associated progenitors from a 330 day old mouse. Cells from the left Gast muscle are depicted in green and from the and right Gast in red; cells from the right Masseter are depicted in purple. MSCs are depicted in brown. Myofiber-associated cells were significantly clustered according to the muscle they were extracted from with the p-values denoted in the figure.

To elucidate the development and migration paths of myofiber-associated progenitors harbored in distinct muscles we performed lineage analyses comparing clones extracted from the Masseter and Gast muscles. Our data revealed that cells are clustered in a hierarchical manner, first according to the muscle from which they originated and then according to their myogenic or non-myogenic type (Figure 4). For example, as shown in Figure 4, clones from the Masseter were significantly clustered; suggesting they share the same ancestor (p = 7.6e-8), and clones from the right and left Gast were clustered as well implying that they share their own ancestor (p = 9.7e-8, and p = 1.3e-5 respectively). Similar results from M2 are presented in Figure S3. These results suggest that each of the tested muscles was populated with distinct sets of ancestors, when each set gave rise to the muscle-specific resident myofiber-associated progenitors. In other words, our results imply that during development, precursors migrate to individual muscles where they further proliferate.

**Figure 5:**
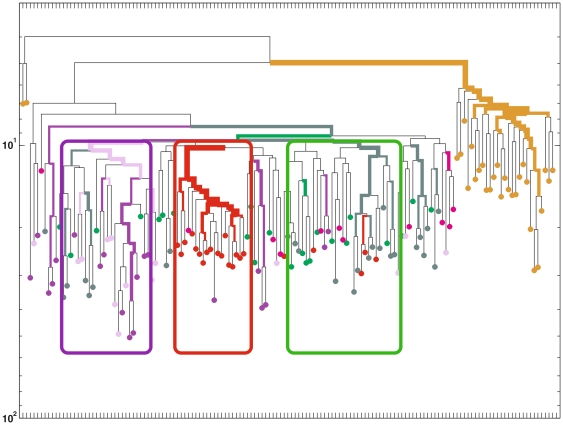
Clustering of MSCs and myofiber-associated myogenic and non-myogenic clones from a 330 days old mouse. The following 7 sets of cells were significantly clustered: MSC (brown), myogenic clones from the Gast left (dark green), non-myogenic clones from Gast left (bright green), myogenic clones from the right Gast (red), non-myogenic clones from the right Gast (pink), myogenic clones from the right Masseter (dark purple) and non-myogenic clones from the right Masseter (bright purple). Purple red and green boxes mark the different clustering by muscles (as in Figure 4).

We next analyzed the reconstructed lineage trees of the myogenic and non-myogenic clones extracted from all tested muscles. For this we compared 6 sets of myofiber-associated cells: left Gast myogenic and non-myogenic, right Gast myogenic and non-myogenic, and right Masseter myogenic and non-myogenic. Data revealed that (i) the myogenic clones cluster separately from non-myogenic clones within the same muscle (Figure 5) and (ii) each of these sets (myogenic, non–myogenic) was clustered individually. This demonstrates that each set of cells evolved from different precursors, meaning that myogenic and non-myogenic clones from a specific muscle are closer to each other than to myogenic (or non-myogenic) clones each from a different muscle. Moreover, combining these results with our findings that there is a muscle-specific clustering of myofiber-bound progenitors implies that during development muscle-bound stem cells first migrate to individual muscles and then differentiate into myogenic and non-myogenic progenitors.

### Do individual myofibers carry adherent cells?

As detailed above, our data show that in addition to myogenic progenitors, myofibers also harbor non-myogenic progenitors of a distinct, yet close, lineage. To shed light on the question whether the non-myogenic progenitors are cells that adhere to the outer surface of myofibers' basal lamina (i.e., adherent cells) we performed an experiment that is detailed in the Supplemented Materials (see Figure S4 and Video S1). In this experiment we determined the position of nuclei (visualized by DAPI incorporation – blue staining) in relation to the basal lamina (immunostained with anti-laminin – red staining) of individual myofibers. When all nuclei were situated beneath the basal lamina – the myofiber was considered free of adherent cells, if nuclei were detected outside the myofiber basal lamina these cells were defined as adherent. Data showed that: (1) over 80% of myofibers were free of adherent cells; and (2) most of the myofibers that did carry adherent cells did not carry more than one such cell. Considering the fact that almost every myofiber analyzed in our experiments contained cells that gave rise to non-myogenic clones (typically- a couple of clones), these data suggest that adherent cells may not be the sole, or even the main, source, for the myofiber associated non-myogenic progenitors (Figure S4 and Video S1).

## Discussion

In the present study we analyzed the lineal relations between cells associated with myofibers and MSCs. MSCs were cloned from the bone marrow and myogenic and non-myogenic cells were cloned from individual myofibers. Our analyses suggest, as depicted in Figure 1D, that primordial stem cells that are the common ancestors of myofiber-associated myogenic and non-myogenic stem cells, first migrate to individual muscles and then part into myogenic and non-myogenic precursors. The latter two populations have a similar, but not identical, developmental path which is different from the lineage of bone marrow MSCs.

Although muscles are known to contain MSCs as part of their interstitium, and determining their lineage relations with myofiber-associated progenitors would have been informative, doing so in our current experimental setting was not practical, as isolating these MSCs requires digesting many muscles [34], and therefore we included in the lineage analysis bone marrow MSCs instead. Determining the lineage relations between bone-marrow MSCs and muscle-adhering MSCs, as well as the lineage relations between the latter and muscle-fiber derived progenitor cells remains a challenge for future research. Still, we note that: (i) MSCs from virtually all post-natal organs and tissues, including muscle interstitium, share very similar characteristics [32]; and (ii) bone-marrow contains circulating MSC cells that reach other/distant organs in the body [33].

Satellite cells (all or a subpopulation) as well as several other types of cells, such as MSCs or white fat cells were suggested to account for such non-myogenic cells [26], [42], [43]. The most attractive candidates are the MSCs as they are present in the muscle tissue and were shown to give rise to cells of several lineages including adipocytes and myoblasts [27], [28], [44] Additionally, by means of multiple immuno-staining of clones derived from mice myofibers, we previously showed that the composition of the non-myogenic clones resembles that of MSC progeny [20]. Our data clearly show that myofibers harbor myogenic and non-myogenic progenitors with a similar (but not identical) developmental path that is significantly different than the lineage of MSCs.

Potential candidate cells for a shared ancestor of the myogenic and non-myogenic founders maybe the mesoangioblasts/pericytes. Pericytes are cells that surround endothelial cells in capillaries and microvessels and are thought to include progenitors of different cell types including skeletal muscle cells [11], [45]. Specifically, Dellavalle and colleagues [46] indicated that pericytes are myogenic precursors, distinct from satellite cells, which are associated with the microvascular walls in human skeletal muscles. Authors also pointed that these cells may represent a correlate of embryonic mesoangioblasts. In another study mesoangioblasts were isolated from embryonic dorsal aorta and shown to participate in postnatal muscle myogenesis [47]. Albeit we cannot rule out the possibility that mesoangioblasts/pericytes are the common ancestors of the founder of MA-NM and MA-M, our past and present studies do not favor this explanation for the following three reasons. First, unlike cultures of pericytes/mesoangioblasms that possess extensive myogenic capacity in-vivo and in-vitro, the MA-NM clones did not contain myogenic cells even after long time culture and albeit supplemented in growth medium that favors myogenesis [20] or upon co-culture with myogenic cells (Shefer, G. and Yablonka-Reuveni, Z unpublished data). Second, recent studies suggest that MSCs are of pericyte origin [11] whereas present results indicate that MA-NM and MA-M founders share a common ancestor which is different from that of the MSCs. Third, if accepting that pericytes are the common ancestor of the MA-NM and MA-M founder, it would have been expected that MA-NM cells from the masseter would have been of a very different lineage than MA-NM from the limb muscles. This is because pericytes in the cephalic region are derived from neural-ectoderm, and not from mesoderm. Nevertheless, our results do not point to a significant lineage difference between clones from the masseter to clones derived from the limb muscles.

Whether the primordial stem cells or the myogenic progenitors represent the satellite cells pool cannot be conclusively established based only on the current lineage analyses. If the primordial stem cells represent the satellite cell population, then upon their population of target muscles they give rise to myogenic and non-myogenic cells. The notion that satellite cells can give rise to non-myogenic cell types accords with studies with rat and mice myofibers [18], [19], [20], [22] and a study with newts [23]. In the latter study, an *in-vivo* approach was taken to determine the fate of satellite cells during limb regeneration after amputation. Data showed that some of the re-introduced labeled satellite-cell derived clones adopted non-myogenic fates. Alternatively, if the myofiber-associated myogenic progenitors represent the satellite cells pool, then satellite cells are homogenous with regards to their differentiation breadth and are only in close, but not same, lineal relations with the ancestors of non-myogenic cells. This accords with evidence implying that satellite cells are uni-potent cells, giving rise to myogenic cells only [48], [49].

Regardless of their origin, the finding that muscle fibers consistently harbor a distinct lineage of non-myogenic stem cells immediately raises the question on the biological function of such cells in health and disease. One may speculate that the presence of non-myogenic progenitors within the muscle maybe of advantage. For example, there may be an advantage in the immediate availability of fibroblasts in case of injury as such cells are needed to synthesize extracellular matrix proteins that take part in scar formation which is necessary for adequate myofiber-repair [50], [51]. The down side of an intimate source of non-myogenic cell may be reveled when the muscle niche is disturbed and encourages the proliferation of non-myogenic rather than the proliferation of myogenic cells. In such cases enhanced proliferation of non-myogenic cells may account for the fibrosis and adipose accumulation characteristics of myopathic diseases and aging [22], [52], [53].

In any event, this current study clearly demonstrates that myofiber-associated non-myogenic progenitors are not the progeny of MSCs. We thus conclude that at least some of the inter-muscular adipocytes and/or fibroblasts are the progeny of myofiber-associated progenitors rather than MSCs. This does not necessarily contradict with recent findings that MSCs also contribute to adipogenesis and fibrogenesis in skeletal muscles [27], [28]. Considering the finding that myogenic and non-myogenic progenitors share similar developmental path we postulate that cells of different source such as fat cells [54], [55] cannot be the source of all non-myogenic cells that develop in myofiber cultures.

In summary, the combination of computation and biological approaches allowed analyzing three different cell types at the same time, a commodity that is not available by any other means up to date. This allowed better understanding the differentiation dynamics of the pools of these stem cells and the lineal relationships between the subpopulations.

## Materials and Methods

### Animals

Mlh1+/−C57Bl/6 (obtained from Michael Liskay) [56] and Mlh1+/− 129SvEv (provided by Ari Elson) were mated to yield Mlh1−/− progeny of the dual backgrounds that served in the present experiments. Two male mice were genotyped as Mlh−/− and sacrificed at the ages of 330 and 44 days (see Table 1). Animal husbandry, maintenance and euthanasia procedures were performed in accordance with the Institutional Animal Care and Use Committee at the Weizmann Institute of Science (IACUC from 15.10.2009, valid till 18.10.2011, Application Num. 04730909-3. The Bio-Ethics Committee of the Weizmann Institute of Science specifically approved this study).

**Table 1:** Age and type of cells extracted for each mouse.

	M1	M2
Age	330 days	44 days
Myofiber-associated cells	116 cells	175 cells
MSC	29 cells	26 cells

### Myofiber isolation and cell cloning

Myofiber association cells were isolated and cultured as described [20] with the modifications as detailed bellow. Briefly, fresh myofibers, which served for clonal analysis of myofiber-associated cells, were isolated from the right and left hindlimb Gast and mastication Masseter. Muscles were digested in 0.2% (w/v) collagenase type I (Sigma-Aldrich) in Dulbecco's Modified Essential Medium (DMEM; high glucose, with L-glutamine, 110 mg/l sodium pyruvate, and pyridoxine hydrochloride; fortified with50 U/ml penicillin and 50 mg/ml streptomycin; GIBCO) for 90 minutes at 37°C [57]. Following digestion, each muscle was rinsed first in BPS and then by 3 sequential transferring to four 100 mm dishes, each containing 7 mL of DMEM. The purpose of these 4 rounds of rinses was to clean the muscle bulk from adherent cells, from loose connective tissue or remains of blood. Muscle was then triturated with a wide-bore pipette to release single myofibers. Every five individual myofibers were transferred to a separate 60 ml dish containing 5 mL of DMEM. The myofibers were swirled and transferred to a second and third DMEM containing 60 ml dishes. The purpose of these 3 rounds of rinses was to minimizing the contribution of non-myogenic cells that are released from the muscle bulk in the process of enzymatic digestion. A short video clip of a 3-D reconstruction of a myofiber showing a segment of myofiber that is free of adherent cells is available at the supplemented material (Video S1). After the three rinses every single myofiber was transferred to a tube containing 1 ml DMEM. Single myofibers, in 1 mL of DMEM, were triturated using a 20 G needle mounted onto a 1 ml syringe. This was done in order to disengage myofiber-associated cells. To obtain cultures at clonal density (i.e., no more than 1 cell per culture) equal volumes of the fiber suspension (i.e., 42 microliters per well) was then equally dispensed into each of the 24 Matrigel pre-coated wells. This was based on previous studies showing that the average number of satellite cells per myofiber of young mice is 2–3 in Masseter [58] and about 24 in young rats [59]. Clones were observed daily for the first 3 days to assure that only one cell (or no cell) in found per well. From the 4^th^ day cultures were inspected every other day for up to 14 days, a stage by which myogenic clones typically develop myotubes. As previously described [20], some clones developed to be myogenic (i.e., clones that contained myotubes) and others were non-myogenic (i.e., clones that were composed of fibroblast-like cells and were absent of myotubes). Molecular characteristics of myogenic and non-myogenic cells that develop in myofiber cultures and in clones were extensively studied by us [20], [60].

### Isolation and culture of mesenchymal stem cells (MSCs)

MSCs were isolated and cultured as described and extensively characterized by other and by us in a series of studies [61], [62], [63]. Briefly, femur bones were cleaned of the soft tissue and epiphysis to allow collection of bone marrow cells (BMC). BMC were flushed out with DMEM using syringe with 21G needle. BMC suspensions were mixed with medium and centrifuged, cell pellets were washed. Cells were counted and diluted in DMEM supplemented with 10% FCS to a concentration of 2.5×10^6^ cells/ml medium. This amount of cells was seeded per well in 24 well dished. After 2–3 day incubation, wells were carefully washed to remove non-adherent cells and then supplemented with fresh culture medium. These selective conditions allowed only adherent fibroblastic cells to develop. Initial colonies were evident within 7–10 days and were followed every other day. These colonies were addressed as single Colony forming unit-fibroblast (CFU-F). Cells were allowed to proliferate for 14 days, before harvest and DNA isolation.

### Whole Genome amplification (WGA) of single cells

WGA was performed using the Illustra GenomiPhi V2 DNA Amplification kit (GE Healthcare Life Sciences, Piscataway, NJ, USA) according to the manufacturer's instructions as described by G. Kumar et al [64]. Briefly, cultured clones transferred to PCR tubes (0.2 ml volume) using 3 µl sample buffer from the kit. In the optimized protocol, 1.5 µl cell-lysis solution (600 mM KOH, 10 mM EDTA, 100 mM dithiothreitol (DTT)) was added to each culture. Cell lysis was carried out for 10 min at 30°C, followed by the addition of 1.5 µl neutralizing solution (4 vol 1 M Tris-HCl, pH 8.0, added to 1 vol 3 M HCl). WGA reaction was initiated by the addition, of 4 µl sample buffer, 9 µl reaction buffer, and 1 µl enzyme mixture, all supplied with the kit. The amplification was then carried out at 30°C for 4 h followed by heat inactivation at 65°C for 10 min. The WGA product was diluted 1∶20 in double distilled water (DDW) and used directly without any further purification as template for subsequent 128 PCRs (Table S1). PCR repeats and negative controls (DDW) were included in every PCR plate. Loci that exhibit a signal in the negative control were excluded from the analysis of all samples run on the corresponding PCR plate. Signal to noise ratio, introduced by the PCR amplification has been assessed for each tree (Figure S1).

### Tree and depth reconstruction

Microsatellite length was analyzed based on the capillary signals. Capillary signals that displayed more than one allele per locus were removed from the analysis. Only cells in which more than 25 alleles were amplified were included in the analysis. Trees were reconstructed using the distance-based neighbor-joining algorithm [65].

Pairs of cells were sequentially merged according to a distance matrix of lineage distances. Each entry in the distance matrix is taken as the maximum likelihood estimate of the number of divisions separating the two cells. The mutation step model and the mutation rates were estimated from the ex-vivo trees. Depth was read off the trees as the branch lengths leading from the root to each terminal leaf. Root signature was taken as the allele size values of tail normal cells (which represent a wide variety of cell types).

### Statistical analysis

P-values for the difference in distributions were calculated using Kolmogorov-Smirnov method. Hypergeometric tests were carried out for each internal branch to assess whether sub tree leafs are enriched for a cell population. P-values declared as significant are corrected for multiple hypothesis testing using false discovery rate of 0.2. Whenever sub trees were embedded only those with the most significant p-value are retained.

## Supporting Information

Figure S1 **PCR repeats, in a 330 (A) days old mouse and 44 (B) days old mouse.** Light blue nodes (•) indicate PCR repeats pairs with close genetic distance. Axis represents depth in arbitrary units.
(TIF)

Figure S2 **Lineage tree of 178 Myofiber-associated cells (blue), and 28 MSC (brown) of a 44 days old mouse.** Each terminal node (blue • or red •) represents a single sampled cell. The vertical axis represents the number of divisions a cell underwent since the zygote, i.e., cell depth. Blue and brown lines indicate significant clustering of myofiber-associated cells and MSCs, in distinct subtrees with a p value<1e-21.
(TIF)

Figure S3 **Clustering of MSC and myofiber-associated cells of 44 days old mouse.** Cells from the left Gast muscle are depicted in green and from the right Gast in red; cells from the right Masseter are depicted in purple. MSCs are depicted in brown. Myofiber-associated cells were significantly clustered according to the muscle they were extracted from with the p values denoted in the figure.
(TIF)

Figure S4 **A Gast myofiber and its myonuclei.** Laminin, that is part of the basal lamina, is shown in red and nuclei, visualized based on DAPI (4′,6-diamidino-2-phenylindole) incorporation, in blue. All nuclei in this segment of the myofiber are situated beneath the basal lamina of the myofiber.
(TIF)

Table S1 **Microsatellite panel.** Different microsatellites loci and sequence used in this paper. Name = Loci name, Color =  fluorescent colors ABI dyes: B = FAM Blue; Y =  NED Yellow; R =  PET Red; G = VIC Green. LIZ Orange was used as a size standard. fwd/rev primer are sequences of the primers used. #repeats is number of repeats in the micro sattelite.
(DOC)

Video S1 **Reconstruction of a three-dimensional (3D)- fluorescent model of the myofiber basal lamina (red) and its associated nuclei (blue).**
(WMV)

## References

[1] Brand-Saberi B, Christ B (2000). Evolution and development of distinct cell lineages derived from somites.. Curr Top Dev Biol.

[2] Huang R, Christ B (2000). Origin of the epaxial and hypaxial myotome in avian embryos.. Anat Embryol (Berl).

[3] Brent AE, Tabin CJ (2002). Developmental regulation of somite derivatives: muscle, cartilage and tendon.. Curr Opin Genet Dev.

[4] Stockdale FE, Nikovits W, Christ B (2000). Molecular and cellular biology of avian somite development.. Dev Dyn.

[5] Gros J, Manceau M, Thome V, Marcelle C (2005). A common somitic origin for embryonic muscle progenitors and satellite cells.. Nature.

[6] Relaix F, Rocancourt D, Mansouri A, Buckingham M (2005). A Pax3/Pax7-dependent population of skeletal muscle progenitor cells.. Nature.

[7] Schienda J, Engleka KA, Jun S, Hansen MS, Epstein JA (2006). Somitic origin of limb muscle satellite and side population cells.. Proc Natl Acad Sci U S A.

[8] Harel I, Nathan E, Tirosh-Finkel L, Zigdon H, Guimaraes-Camboa N (2009). Distinct origins and genetic programs of head muscle satellite cells.. Dev Cell.

[9] Pouget C, Pottin K, Jaffredo T (2008). Sclerotomal origin of vascular smooth muscle cells and pericytes in the embryo.. Dev Biol.

[10] Corselli M, Chen CW, Crisan M, Lazzari L, Peault B (2010). Perivascular ancestors of adult multipotent stem cells.. Arterioscler Thromb Vasc Biol.

[11] Crisan M, Yap S, Casteilla L, Chen CW, Corselli M (2008). A perivascular origin for mesenchymal stem cells in multiple human organs.. Cell Stem Cell.

[12] Friedenstein AJ, Chailakhjan RK, Lalykina KS (1970). The development of fibroblast colonies in monolayer cultures of guinea-pig bone marrow and spleen cells.. Cell Tissue Kinet.

[13] Friedenstein AJ, Petrakova KV, Kurolesova AI, Frolova GP (1968). Heterotopic of bone marrow. Analysis of precursor cells for osteogenic and hematopoietic tissues.. Transplantation.

[14] Owen M (1988). Marrow stromal stem cells.. J Cell Sci.

[15] Mauro A (1961). Satellite cell of skeletal muscle fibers.. J Biophys Biochem Cytol.

[16] Collins CA, Olsen I, Zammit PS, Heslop L, Petrie A (2005). Stem cell function, self-renewal, and behavioral heterogeneity of cells from the adult muscle satellite cell niche.. Cell.

[17] Asakura A, Rudnicki MA, Komaki M (2001). Muscle satellite cells are multipotential stem cells that exhibit myogenic, osteogenic, and adipogenic differentiation.. Differentiation.

[18] Csete M, Walikonis J, Slawny N, Wei Y, Korsnes S (2001). Oxygen-mediated regulation of skeletal muscle satellite cell proliferation and adipogenesis in culture.. J Cell Physiol.

[19] Rossi CA, Pozzobon M, Ditadi A, Archacka K, Gastaldello A (2010). Clonal characterization of rat muscle satellite cells: proliferation, metabolism and differentiation define an intrinsic heterogeneity.. PLoS One.

[20] Shefer G, Wleklinski-Lee M, Yablonka-Reuveni Z (2004). Skeletal muscle satellite cells can spontaneously enter an alternative mesenchymal pathway.. J Cell Sci.

[21] Wada MR, Inagawa-Ogashiwa M, Shimizu S, Yasumoto S, Hashimoto N (2002). Generation of different fates from multipotent muscle stem cells.. Development.

[22] Brack AS, Conboy MJ, Roy S, Lee M, Kuo CJ (2007). Increased Wnt signaling during aging alters muscle stem cell fate and increases fibrosis.. Science.

[23] Morrison JI, Borg P, Simon A (2010). Plasticity and recovery of skeletal muscle satellite cells during limb regeneration.. FASEB J.

[24] Buckingham M, Bajard L, Chang T, Daubas P, Hadchouel J (2003). The formation of skeletal muscle: from somite to limb.. Journal of Anatomy.

[25] Tajbakhsh S (2003). Stem cells to tissue: molecular, cellular and anatomical heterogeneity in skeletal muscle.. Current Opinion in Genetics & Development.

[26] Shefer G, Yablonka-Reuveni Z (2007). Reflections on lineage potential of skeletal muscle satellite cells: do they sometimes go MAD?. Crit Rev Eukaryot Gene Expr.

[27] Joe AW, Yi L, Natarajan A, Le Grand F, So L (2010). Muscle injury activates resident fibro/adipogenic progenitors that facilitate myogenesis.. Nat Cell Biol.

[28] Uezumi A, Fukada S, Yamamoto N, Takeda S, Tsuchida K (2010). Mesenchymal progenitors distinct from satellite cells contribute to ectopic fat cell formation in skeletal muscle.. Nat Cell Biol.

[29] Meirelles LDS, Chagastelles PC, Nardi NB (2006). Mesenchymal stem cells reside in virtually all post-natal organs and tissues.. Journal of Cell Science.

[30] Alm JJ, Koivu HM, Heino TJ, Hentunen TA, Laitinen S (2010). Circulating plastic adherent mesenchymal stem cells in aged hip fracture patients.. J Orthop Res.

[31] Frumkin D, Wasserstrom A, Itzkovitz S, Stern T, Harmelin A (2008). Cell lineage analysis of a mouse tumor.. Cancer Res.

[32] Frumkin D, Wasserstrom A, Kaplan S, Feige U, Shapiro E (2005). Genomic variability within an organism exposes its cell lineage tree.. PLoS Comput Biol.

[33] Wasserstrom A, Adar R, Shefer G, Frumkin D, Itzkovitz S (2008). Reconstruction of cell lineage trees in mice.. PLoS One.

[34] Wasserstrom A, Frumkin D, Adar R, Itzkovitz S, Stern T (2008). Estimating cell depth from somatic mutations.. PLoS Comput Biol.

[35] Reizel Y, Chapal-Ilani N, Adar R, Itzkovitz S, Elbaz J (2011). Colon stem cell and crypt dynamics exposed by cell lineage reconstruction.. PLoS Genet.

[36] Salipante SJ, Horwitz MS (2006). Phylogenetic fate mapping.. Proc Natl Acad Sci U S A.

[37] Salipante SJ, Horwitz MS (2007). A phylogenetic approach to mapping cell fate.. Curr Top Dev Biol.

[38] Salipante SJ, Thompson JM, Horwitz MS (2008). Phylogenetic fate mapping: theoretical and experimental studies applied to the development of mouse fibroblasts.. Genetics.

[39] Salipante SJ, Kas A, McMonagle E, Horwitz MS (2010). Phylogenetic analysis of developmental and postnatal mouse cell lineages.. Evol Dev.

[40] Barker N, van Es JH, Kuipers J, Kujala P, van den Born M (2007). Identification of stem cells in small intestine and colon by marker gene Lgr5.. Nature.

[41] Reizel Y, Chapal-IIani N, Adar R, Itzkovitz S, Elbaz J (2011). Colon stem cell dynamics exposed by cell lineage analysis.. Plos genetics.

[42] Grounds MD, Yablonka-Reuveni Z (1993). Molecular and cell biology of skeletal muscle regeneration.. Mol Cell Biol Hum Dis Ser.

[43] Young HE, Black AC (2004). Adult stem cells.. Anat Rec A Discov Mol Cell Evol Biol.

[44] Bruder SP, Fink DJ, Caplan AI (1994). Mesenchymal stem cells in bone development, bone repair, and skeletal regeneration therapy.. J Cell Biochem.

[45] Andreeva ER, Pugach IM, Gordon D, Orekhov AN (1998). Continuous subendothelial network formed by pericyte-like cells in human vascular bed.. Tissue Cell.

[46] Dellavalle A, Sampaolesi M, Tonlorenzi R, Tagliafico E, Sacchetti B (2007). Pericytes of human skeletal muscle are myogenic precursors distinct from satellite cells.. Nat Cell Biol.

[47] De Angelis L, Berghella L, Coletta M, Lattanzi L, Zanchi M (1999). Skeletal myogenic progenitors originating from embryonic dorsal aorta coexpress endothelial and myogenic markers and contribute to postnatal muscle growth and regeneration.. J Cell Biol.

[48] Kanisicak O, Mendez JJ, Yamamoto S, Yamamoto M, Goldhamer DJ (2009). Progenitors of skeletal muscle satellite cells express the muscle determination gene, MyoD.. Dev Biol.

[49] Starkey JD, Yamamoto M, Yamamoto S, Goldhamer DJ (2011). Skeletal muscle satellite cells are committed to myogenesis and do not spontaneously adopt nonmyogenic fates.. J Histochem Cytochem.

[50] Ciciliot S, Schiaffino S (2010). Regeneration of mammalian skeletal muscle. Basic mechanisms and clinical implications.. Curr Pharm Des.

[51] Kaariainen M, Jarvinen T, Jarvinen M, Rantanen J, Kalimo H (2000). Relation between myofibers and connective tissue during muscle injury repair.. Scand J Med Sci Sports.

[52] Bernasconi P, Torchiana E, Confalonieri P, Brugnoni R, Barresi R (1995). Expression of transforming growth factor-beta 1 in dystrophic patient muscles correlates with fibrosis. Pathogenetic role of a fibrogenic cytokine.. J Clin Invest.

[53] Wagner KR (2008). Approaching a new age in Duchenne muscular dystrophy treatment.. Neurotherapeutics.

[54] Seale P, Kajimura S, Spiegelman BM (2009). Transcriptional control of brown adipocyte development and physiological function–of mice and men.. Genes Dev.

[55] Shepard JL, Zon LI (2000). Developmental derivation of embryonic and adult macrophages.. Curr Opin Hematol.

[56] Baker SM, Plug AW, Prolla TA, Bronner CE, Harris AC (1996). Involvement of mouse Mlh1 in DNA mismatch repair and meiotic crossing over.. Nat Genet.

[57] Shefer G, Yablonka-Reuveni Z (2005). Isolation and culture of skeletal muscle myofibers as a means to analyze satellite cells.. Methods Mol Biol.

[58] Ono Y, Boldrin L, Knopp P, Morgan JE, Zammit PS (2010). Muscle satellite cells are a functionally heterogeneous population in both somite-derived and branchiomeric muscles.. Dev Biol.

[59] Shefer G, Carmeli E, Rauner G, Yablonka-Reuveni Z, Benayahu D (2008). Exercise running and tetracycline as means to enhance skeletal muscle stem cell performance after external fixation.. J Cell Physiol.

[60] Shefer G, Benayahu D (2010). SVEP1 is a novel marker of activated pre-determined skeletal muscle satellite cells.. Stem Cell Rev.

[61] Benayahu D, Fried A, Zipori D, Wientroub S (1991). Subpopulations of marrow stromal cells share a variety of osteoblastic markers.. Calcif Tissue Int.

[62] Liu Z, Graff E, Benayahu D (2000). Effect of raloxifene-analog (LY 117018-Hcl) on the bone marrow of ovariectomized mice.. J Cell Biochem.

[63] Kuznetsov SA, Krebsbach PH, Satomura K, Kerr J, Riminucci M (1997). Single-colony derived strains of human marrow stromal fibroblasts form bone after transplantation in vivo.. J Bone Miner Res.

[64] Kumar G, Garnova E, Reagin M, Vidali A (2008). Improved multiple displacement amplification with phi29 DNA polymerase for genotyping of single human cells.. Biotechniques.

[65] Saitou N, Nei M (1987). The neighbor-joining method: a new method for reconstructing phylogenetic trees.. Mol Biol Evol.

