# Supplementary figures and images for: Muscle-Bound Primordial Stem Cells Give Rise to Myofiber-Associated Myogenic and Non-Myogenic Progenitors
Source: PLoS One. 2011 Oct 14;6(10):e25605. doi: 10.1371/journal.pone.0025605 (PMC3194814; doi:10.1371/journal.pone.0025605)

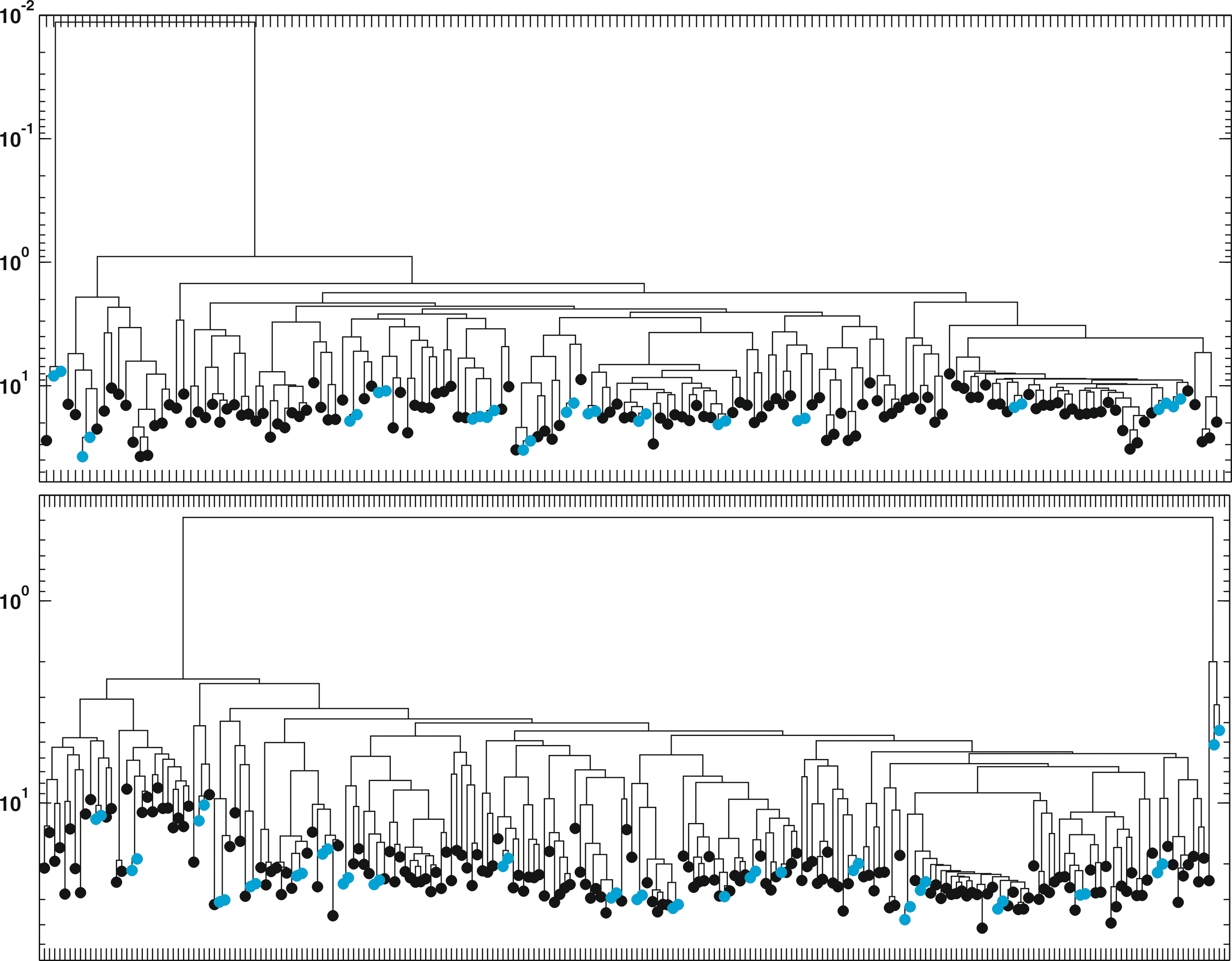

Supplement: Figure S1 — PCR repeats, in a 330 (A) days old mouse and 44 (B) days old mouse. Light blue nodes (•) indicate PCR repeats pairs with close genetic distance. Axis represents depth in arbitrary units. (TIF) [file pone.0025605.s001.tif]

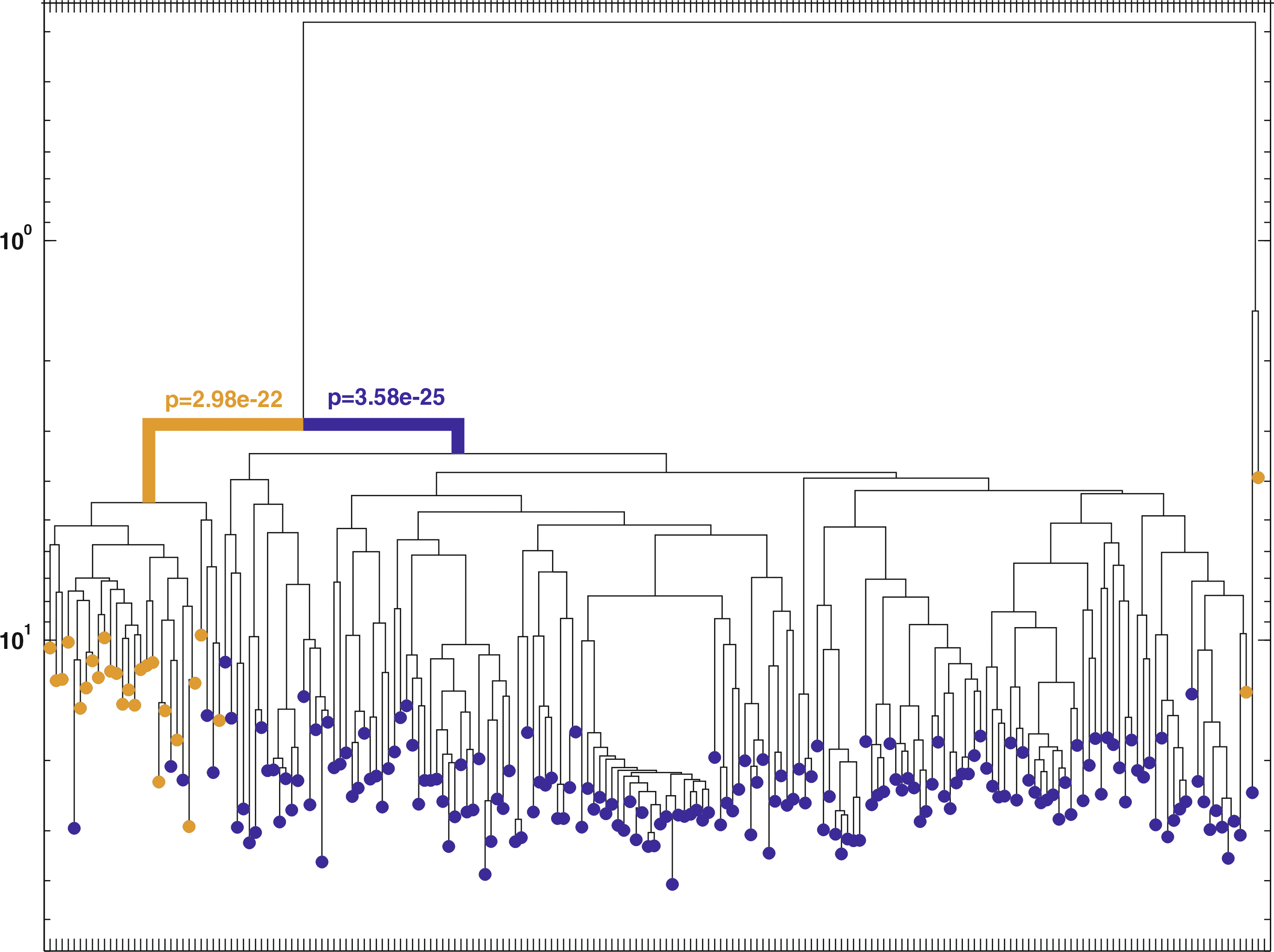

Supplement: Figure S2 — Lineage tree of 178 Myofiber-associated cells (blue), and 28 MSC (brown) of a 44 days old mouse. Each terminal node (blue • or red •) represents a single sampled cell. The vertical axis represents the number of divisions a cell underwent since the zygote, i.e., cell depth. Blue and brown lines indicate significant clustering of myofiber-associated cells and MSCs, in distinct subtrees with a p value<1e-21. (TIF) [file pone.0025605.s002.tif]

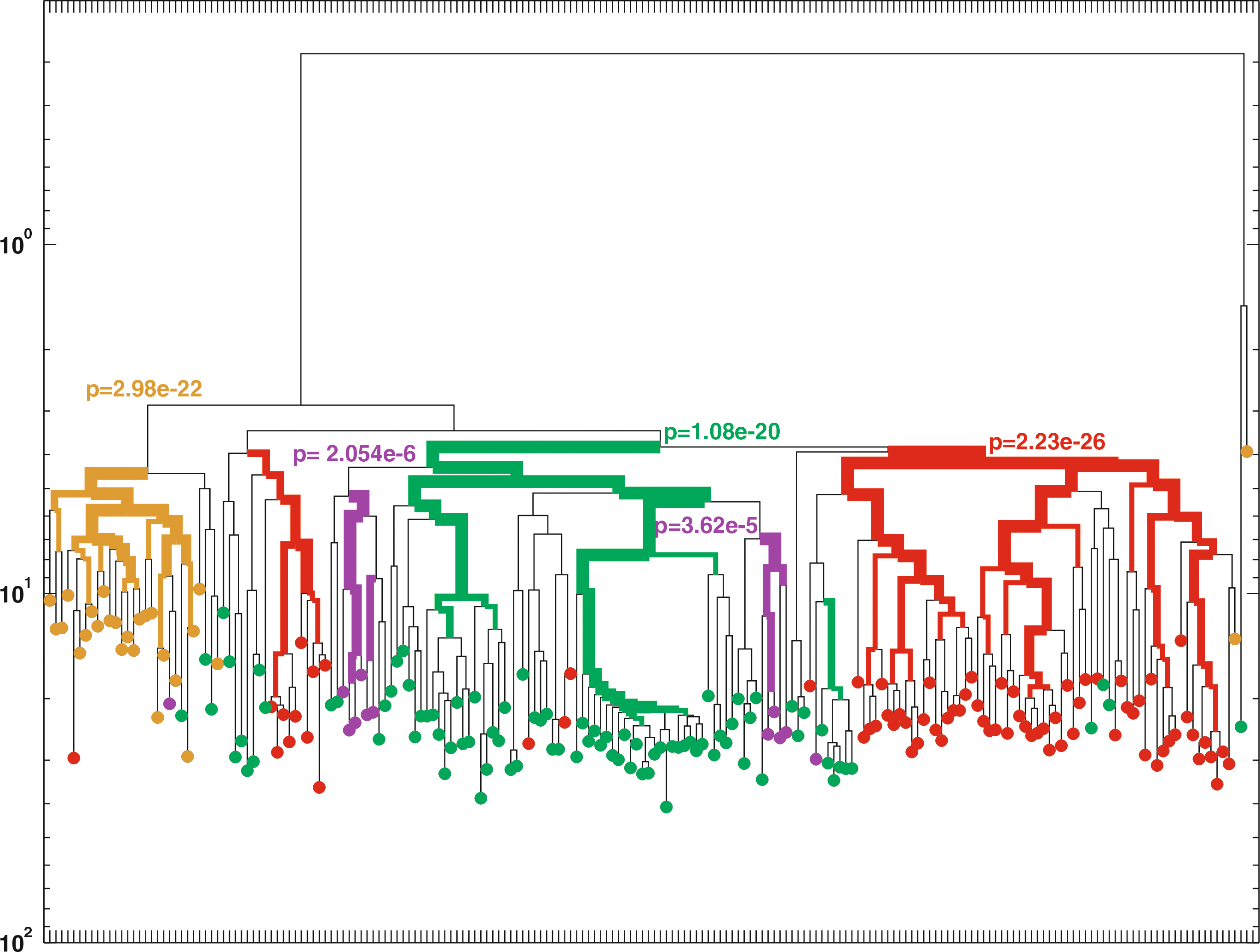

Supplement: Figure S3 — Clustering of MSC and myofiber-associated cells of 44 days old mouse. Cells from the left Gast muscle are depicted in green and from the right Gast in red; cells from the right Masseter are depicted in purple. MSCs are depicted in brown. Myofiber-associated cells were significantly clustered according to the muscle they were extracted from with the p values denoted in the figure. (TIF) [file pone.0025605.s003.tif]

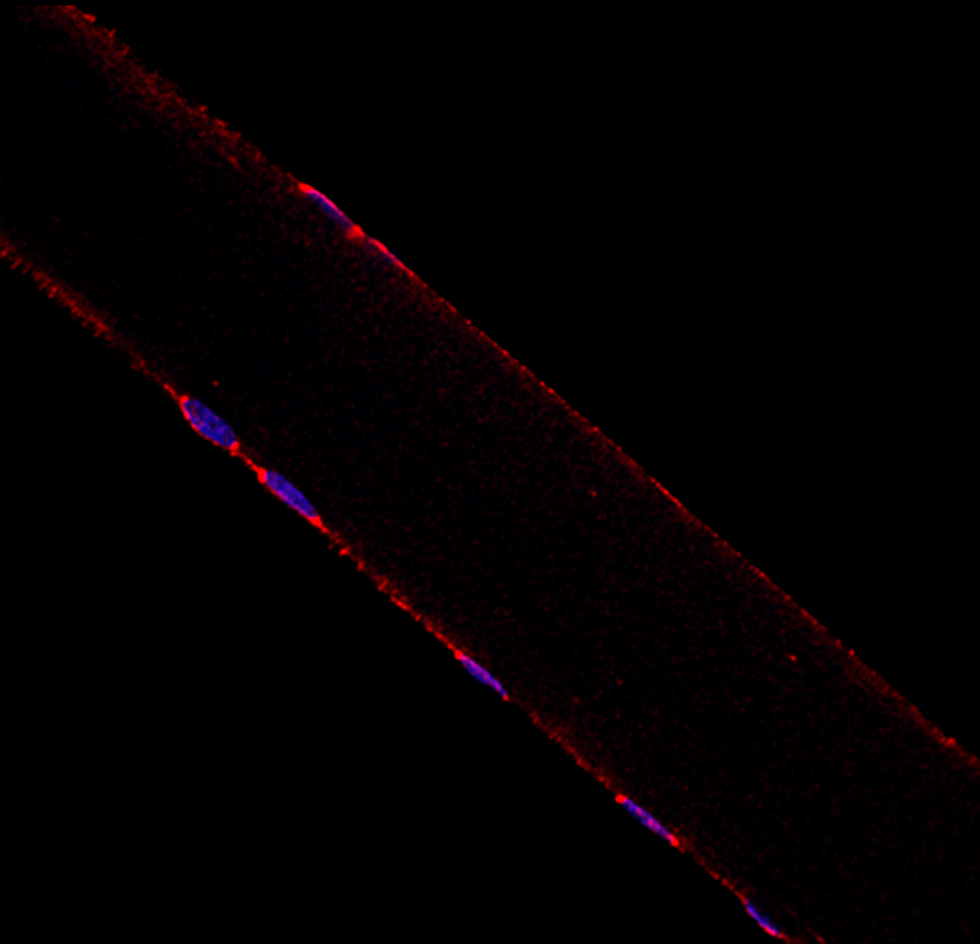

Supplement: Figure S4 — A Gast myofiber and its myonuclei. Laminin, that is part of the basal lamina, is shown in red and nuclei, visualized based on DAPI (4′,6-diamidino-2-phenylindole) incorporation, in blue. All nuclei in this segment of the myofiber are situated beneath the basal lamina of the myofiber. (TIF) [file pone.0025605.s004.tif]
