# Supplementary material for: Muscle-Bound Primordial Stem Cells Give Rise to Myofiber-Associated Myogenic and Non-Myogenic Progenitors
Source: PLoS One. 2011 Oct 14;6(10):e25605. doi: 10.1371/journal.pone.0025605 (PMC3194814; doi:10.1371/journal.pone.0025605)
Supplement: Table S1 — Microsatellite panel. Different microsatellites loci and sequence used in this paper. Name = Loci name, Color = fluorescent colors ABI dyes: B = FAM Blue; Y = NED Yellow; R = PET Red; G = VIC Green. LIZ Orange was used as a size standard. fwd/rev primer are sequences of the primers used. #repeats is number of repeats in the micro sattelite. (DOC) [file pone.0025605.s005.doc]

| Name | Color | chr | fwd primer | rev primer | # repeats |
| --- | --- | --- | --- | --- | --- |
| mX1_T32 | B | chrX | CCACAGAGCAACTGAAATGAGG | TCCCTCGATGCCACTAGAACAC | 32 |
| mX2_A43 | B | chrX | ACAGGGGAAGGGGAAACAACC | GGTTTCCCACTTGTAGAACAGTTTGC | 43 |
| mX3_AC30 | B | chrX | TCCTGTTAAGAGACCATCACAGTGG | TCCAAATAGGCATTTGATCACCTC | 30 |
| mX8_GA35 | B | chrX | TTCTGACCACTAGCTACAACACTGCTC | GAGAAGAAAACTGATCTCCCAAAGTCC | 35 |
| mX37_AG32 | G | chrX | GGTGACCATTTCTCTGCTTGTTTC | TGTTCTAAAGATCGACTGCACACAG | 32 |
| mX34_T38 | G | chrX | CATATTAACCAGGCCCCAGGAC | GAACAAAAGGCCCCTAGAAGATG | 38 |
| mX31_TC35 | G | chrX | GCATGACTTGGACAACTGCTTTC | GCTTCTGGGTCAAGGGAATGTG | 35 |
| mX64_TC36 | G | chrX | GGGGAGGATCTTCTGGCTCTG | GGCAAGGCACCACAACATGAG | 36 |
| mX25_T34 | Y | chrX | TGAAACAACATCTTGCCAATCC | GTTCTTTGAACAACTGGGAAATTATTG | 34 |
| mX30_A31 | Y | chrX | CAACTGCAGTGGGCTCACCAG | AGCCATGAGTCCCTTTGTGGAG | 31 |
| mX111_GA29 | Y | chrX | ATCCCCCTTGTTTTTCCCTCTG | TTCGAAGGTTTCAGTCAAATATCAGTC | 29 |
| X20 | Y | chrX | GGCATCCTCGCTATTCCATGAG | CAAATGCTGTGGAATTCACCAATG | 42 |
| mX13_A45 | R | chrX | TGCTCAAGTATTCACCAAGACATGC | GGAGAGTGTTTTTCGGGGTTTTC | 45 |
| mX10_A32 | R | chrX | ACCCAAAGGGGCAATCTTTTTC | TGGCTACTGAGGTAACCAAATTTCC | 32 |
| L9348 | R | chr18 | gctggtggaattatttgtggaaac | aagccggctcctagtgacttac | 41 |
| M18 | R | chr3 | AGACCAGGCACCACCAGTCAAG | CGTAAAGAACGCAGATAAAGCTTGC | 30 |
| mX65_TC40 | B | chrX | TGACCCAGAGCCAAATAGCAAG | TGGGAATTGGTTCATGGGAAAG | 40 |
| mX122_TC32 | B | chrX | CTTCTTACCTCGCCAGCCTTTC | TGGCTATTTCATGATATGGTTACAGG | 32 |
| mX79_T37 | B | chrX | GGCAGGTCCATTCAGTCTCCTC | TTTTAAGGAGGGAGCCCGGAAC | 37 |
| mX44_A43 | B | chrX | CATTCATCCAGAAATGGTAGTGCATC | GAGCTGTTGCATGTTATGGCTAGG | 43 |
| IDT13 | G | chr2 | GGAGGGTTTTAAATAGGGAATGTGAAG | TGCAAAGTGCCCTTCTTTGACC | 33 |
| X66 | G | chrX | CACCTCATTTCTAGTGAAAGGATTTTG | CCTTCTATGCCAGTTGTGGTAAATG | 34 |
| mX75_A31 | G | chrX | CTCCCCCATTGTTTTGAACAGC | GCGACAACCCTGTCTAGGCAAC | 31 |
| X88 | G | chrX | GGGTGCTGGGATTCTGACTTAGG | TGGGATATGTTTTTGGAAGCATGAC | 32 |
| mX73_AG30 | Y | chrX | AGCCTTTAGCATGAGCTTTATCAGG | CGCCTGTATTTCTCTTAGTGCCCTATC | 30 |
| mX54_TC32 | Y | chrX | TGCCAGAGCTGCCTTGTATGAG | CAGCTCCTTTCCATTGACTGCTC | 32 |
| mX19_T47 | Y | chrX | TCAAATGAAATGCCCCACACTG | CTGTCTGCATTTGGGGAGGTG | 47 |
| mX56_CA30 | Y | chrX | ACCCCAATCCCCGAGGAACTAC | CTACGGCTGGGTTGTTTTGAGC | 30 |
| mX29_A50 | R | chrX | GGCATCAGATTCTAAGGTTGTCG | AGGTTGGAAGCCAATGGAAATC | 50 |
| mX46_GA29 | R | chrX | GGAAAAGTGAGTGAGCATGTGTGG | AGCTGGCCTGCATACATTCACC | 29 |
| mX39_GA30 | R | chrX | TTCCCCAAGAAGATTCCACTGC | GCCCATACCTTGATCTCCCTCTG | 30 |
| mX36_T34 | R | chrX | CGGCAATTGATGCTCTGAATG | CTCCCCCTGCTCCTTCATACAC | 34 |
| mX113_GA36 | B | chrX | CCAGGACAGCTCTATAGCAAATGAATC | TGAGCCCACCACAGGTAGTCAG | 36 |
| X70 | B | chrX | TCCCCCTCTTCTCTCTCTGCTC | TAGGGAGAGGGGATCGCAGAAC | 47 |
| mX135_T41 | B | chrX | AAAAGCAGATTCAGCAGGAGCAG | GAGGGCCCAGCAAACCTTACAG | 41 |
| IDT12 | B | chr4 | GAATAGCATCACCGCACTGCAC | AGAGGTCCGTTGCATCTGTTGG | 33 |
| mX101_A46 | G | chrX | GCCATCTAAAGAAATGTGTGGTTGC | GTTTAGGCCTGGACTGGGACAG | 46 |
| mX126_TC35 | G | chrX | CGCAGCCCTGCACATTCTATAAC | TCCACTCTGATTGCCTCCAGTTC | 35 |
| mX95_AC30 | G | chrX | CACCTGGCCATAATTTCCATACC | TTCTCATCTTCCGTGGCTTGTG | 30 |
| mX24_GA36 | G | chrX | TCAGAGGAGATGAGGGAGAAGAAAG | GGGAAAAAGCAAGGAAATGCTC | 36 |
| mX89_GA32 | Y | chrX | GCCTGCTCTTAAAATGCCATGATAC | TCTCCAGAGGGGGCATACAAAG | 31 |
| mX114_T37 | Y | chrX | CAGTGGCCAAAAATTGTTCCTG | GTGCTAGTGAGGCGAAGGGAAG | 37 |
| mX175_AG33 | Y | chrX | TGCATTATTGAGCAGCACTTCTGAG | GGTTGCTTCATGCCAAGTTCG | 33 |
| mX88_GT29 | Y | chrX | TGTATAGCTAGAGAGGAAACTGGATGG | GCACACAGAAGACTCCCCACAG | 29 |
| mX61_GA33 | R | chrX | GGACCTCTTGGTATGCCCACTG | GAAGAATTTCCCACCTCTAGTCAAGG | 33 |
| mX42_TA29 | R | chrX | TGATGGCACATCACAAGCAATG | CCAGAATGAGGGTGTAGCATGG | 29 |
| mX47_AC34 | R | chrX | GAACATGGGCCTCACCTTTCAC | AGCCTGAGCATTCTCTGCCTTC | 34 |
| mX60_T39 | R | chrX | TGGTAGGATTCCAAGTTGGCAAAG | CAGATCACTGCTTCCCAGCTTTAG | 39 |
| mX129_TC38 | B | chrX | CAGCCCAATTCCCCACAGAAC | GCAATTTGAAAAATGATCGAGACG | 38 |
| mX158_TC30 | B | chrX | TTGCTATGTGGGTGAAGATAGAAGC | CACAGGCATGGTAATAGGATCAGG | 30 |
| mX199_T61 | B | chrX | CATGCAAGCCAGCCTATGTTTG | TGTGTCATCAGATAATGCAATGAGG | 61 |
| mX156_GA29 | B | chrX | ACTGCAAATCACCAAGGAGAGG | AATCAAGGGTTCCAAAGCATGG | 29 |
| mX133_T43 | G | chrX | TGGAGCATGTGACCTCCAGAAG | TCCCCCTCTGGAGAATTGTGAC | 43 |
| L2454 | G | chr14 | TTCCCCACATCGCTGTAAATGG | TGGCCTGAGACAAAAGCCTAGC | 13 |
| mX103_CT39 | G | chrX | CCAGGCCAGGCATGAATACTG | GCAGGTGCATCAAAAATCAAAGG | 39 |
| mX84_A47 | G | chrX | TTCACAAGAGTATGCAGGACATTGC | GTTAGTGGGCTCCCTCCCTCTC | 47 |
| mX121_A39 | Y | chrX | TCACAGAAAAGCTTCTTGGTTAGGC | ACCATTTAGTGGTTTGAAGAAGAAGG | 39 |
| mX90_GA30 | Y | chrX | CCCCTGTATTCATACCTGCATTCC | GGCCGGTCTGTTTAATAAGCTATTTTG | 30 |
| mX187_TG30 | Y | chrX | GGGCAGAAAACAAGGTTTGGAG | GGACATCTCTGATCACCATCTGAAG | 30 |
| mX109_TG35 | R | chrX | TGGGTAGACAAGCTGGCCTGAG | GCTGGGCTGAGTGGAAAGCTAC | 35 |
| mX138_AG32 | R | chrX | TTTCACCTTGGTCCAAACCAATAG | CAAACAAGAAGCTGGCCATTTACAG | 32 |
| mX107_CT30 | R | chrX | GCTGGGAAATCTATGGCAACTG | GGTTTCTGGAACAGCAGGAAGC | 30 |
| mX108_TC35 | R | chrX | TCCATCATAAACAACAGCAATCTCC | TGGGCTCAATATCATTTTTCAAGC | 35 |
| mX193_AG36 | B | chrX | AGGGAGCCCCCTTCTTTTCC | CCCACTCCACGTGCATTAACTC | 36 |
| mX194_T40 | B | chrX | AAGGGGTTTACTGGCCCCTTC | CAGAGGAGAGTAACATTGGCATTGAG | 40 |
| mX183_A36 | B | chrX | AGAGGAGCTGGAGGGAGGAAAG | CCTCAACAGAATCCTGCCACTTC | 36 |
| mX212_TG33 | B | chrX | AAACAGAGCTGGGTGCTGGAAC | TCCATGGGATGCTCAGAAACAG | 33 |
| mX149_A47 | G | chrX | GGCCCTCCTTCTGTACTCTCTCC | TTTGGAGACTTGCCAGAATTGG | 47 |
| mX82_T33 | G | chrX | CACCCATGAAGGGGACAATGAC | AAACCTGCCACAGGGAATATGG | 33 |
| mX191_TC33 | G | chrX | AAGCAAGAGCACATCCCCTGAG | CTCCGAAAGCCGCTTCCTACTC | 33 |
| mX120_T32 | G | chrX | GATGCAAATGTGACCTGGTTTAGC | GCACATAATCCAAAGAAGCCACAAG | 32 |
| mX153_TC33 | Y | chrX | GGTGCTGTAATCTCCCCAAGTCC | CCCACTTCCCATTTCATGTGTG | 33 |
| mX94_AG64 | Y | chrX | CCTTTGTTCTCCTCCCCTTTCC | GAGGGGGAGGCTGGTGACAG | 64 |
| mX99_AT31 | Y | chrX | GCATGAAAGAAAGATGAGAGTGCTTG | GGTGTTCCTTGTATAACCAAGCTGAAG | 31 |
| mX141_T33 | R | chrX | TTGCTGCTGTAGCTGACACTGAG | CACTAAATTCACTGTCCATTGCTTCTG | 33 |
| M7 | R | chr6 | TCCAGCCTTCAGTAGGCACAGG | GGACAACTACCACAAAATTCCAAGG | 36 |
| mX59_A39 | R | chrX | CCACACCTGGCAGAAGTATGAGG | AATCCTGCCTTCCAGGTCCTTC | 39 |
| mX152_A38 | R | chrX | CAATTGCGGTACCACCACAGTC | CAGAGTTGAATGCCCACACCAG | 38 |
| mX209_A53 | B | chrX | CCGAGGATCTTTCCTCGTTTATTG | TTCATGCTGTCCCAGACCAGTG | 53 |
| mX178_A34 | B | chrX | GTGCTCATATGGACAGTGCCATTC | GGTGGAGAAGGCTTATTCATAGAAGAG | 34 |
| mX127_AT31 | B | chrX | TGCATAGACCAGGAATTCTGAAAATG | TCATGTGCCCCTGTTTGTTTTC | 31 |
| mX168_TC34 | B | chrX | TTTTCCCAACCTCTCAAACTGC | AGAAGTGGGGTGAGGGGGATAG | 34 |
| mX213_A59 | G | chrX | TGGGGAGGACAAAAAGTTTAGATGAG | TTTCCTTCAACCTGCAACATCC | 59 |
| mX154_TC31 | G | chrX | TTGATGGGGATTTTACCCTTGC | GAAAGATCCAGTGAATGAGAATCAGG | 31 |
| mX207_T46 | G | chrX | TGTCCTGTGGCACCAAGTATCTTC | GGAAAGGGAGAGGAGGAACAAGAG | 46 |
| X68 | G | chrX | GGGTGAACCAAATCAAACACAGG | TGGCAGCTGGAGAGAAAGATGG | 35 |
| mX169_T37 | Y | chrX | TCTGCCAGAATAATTTGCTCATTCC | CCCACAAACAGCAAGAAACCTG | 37 |
| X51 | Y | chrX | TGAGGTGGATATTTCCTATCCATTCC | TTTCATGGCTTTTAGAACGGTTG | 43 |
| mX171_TC30 | Y | chrX | CAGCTTGGGGCCATCTTAACTG | GCCAAGTGTCAAAGCTGCTTCC | 30 |
| mX188_T35 | Y | chrX | GCTGACGGGAATACAGACCACAC | CCTGTGGCCTTTGCCTTTAATC | 35 |
| mX205_T38 | R | chrX | CATGGGAATTTGCCCAGAAAG | TCTTTTTCTTCATTGCCAGCTCAC | 38 |
| mX146_T37 | R | chrX | CAGCCAAGGCTAAACTGGAACC | CTCCTTTCCAGCCCCAGCTAAC | 37 |
| mX119_TC29 | R | chrX | TGGATTCCGATATTCAACAATACATCC | CCAAATGAGGACTACGGCCAAG | 29 |
| mX172_TC34 | R | chrX | CACAGTGTTTTGATGTTATGCCAAG | CCAACCAACCAAAACAAACAAAC | 34 |
| IDT9 | B | chr13 | GGGCAGCATCAGCATGGTATC | GAGGACCAGTAGGGCAAACCTG | 32 |
| IDT10 | B | chrX | TGCTCTATGGCCACACTCATGTC | TCATTGCCTTTGGCTCACTGAC | 31 |
| X7 | B | chrX | CATGTGAAAGTGGTGTCAACTTGG | CAGTATTTGGTGGCCTTTCATCG | 23 |
| X76 | B | chrX | TGGCCTTGTCATTTACATCTGC | TTAGAATTGGCAGGCTCACACC | 36 |
| D2Mit100 | G | chr2 | GTGTTCCTAAGGTTGTATTTTGGC | GAAATTTGACAATTGCTAGGTGC | 18 |
| IDT7 | G | chr13 | GCCCCTGAATCTTGAACTGGTG | CCCCAAAAGTAGCCAACAGTGG | 32 |
| X63 | G | chrX | GCAAGCAGTTTGAGTCACAAAGG | TCTTTGATAAAGCATTCAGATCTACCC | 37 |
| X12 | G | chrX | TTCTAGTCCATCCAGCCCTTCC | GGGGCGTGCTGTACCTTAATTG | 41 |
| D1Mit1001 | Y | chr1 | TTGTGTGTAGTACAGTGTTGGTGG | TGGTTCCTGACATCAATCTCC | 20 |
| X62 | Y | chrX | CCCCACCCACTATTTTTCTCACC | AATTCTTTTCCTGTCAATTCTTTGTCC | 34 |
| X52 | Y | chrX | GGCATCCTCCACTCTGGGAAAC | TCAAACCCCACCCCATAGAGG | 34 |
| X60 | Y | chrX | TTCTTTGTCTTAAAGTGGGAATGAGG | TGGCATTTGTGGAGGATATCTGG | 45 |
| X45 | R | chrX | TCATGACGTCAGAAATGATCTGC | CCCTATGAGGTACATCACCCAAGC | 32 |
| L9911 | R | chr10 | taggaaggcatggtgctagg | TGCAAGTATGTGAACTTTGAGTTTTC | 51 |
| L3501 | R | chr10 | AAAGCCCCAAGAAAAAGAACAC | TGCCTGCAAATTTCATGATGTC | 45 |
| X77 | B | chrX | CATTTCCTGGAGGGTGGTTCAG | TCTGTTTTCCAATCAGGGGTCTC | 39 |
| X38 | B | chrX | TCTCCCCAGTTCCCATCTAGAGC | TGCTCTCATTGCCTCTCACACC | 29 |
| X67 | B | chrX | TCTCAACATTCGGGATGAGATAAGG | TCCATCCAAGGTCTCCAAGGTG | 32 |
| X24 | B | chrX | CGGGGACATTCCACGTTAGTTC | GCTTATGGTGGATTCCCTGTGC | 47 |
| X69 | G | chrX | TGCCTACTGCCTTTACATGAGGTC | CCAAGGTGAGATTTCATCCTGAGTC | 39 |
| IDT6 | G | chr13 | TGCTCTATGGCCACACTCATGTC | TCATTGCCTTTGGCTCACTGAC | 31 |
| IDT15 | G | chr1 | TCTGGGTTGACGTGTCTCTTGC | AAAAGGGAAGGTGGGAGTGAGG | 33 |
| X65 | Y | chrX | GCTGTGTCAGGAACTCAGCTCTC | CAGATGGCCTGTTTGCTGTCTG | 35 |
| L3464 | Y | chr16 | TTCAGTCTCCTCCCATCTGTGC | CGATGTGTTGTGCATTGGTTCC | 43 |
| X34 | Y | chrX | CAGCAAAAACAGGTGGCTGTG | AATGCAGGGCTCAGGAAATGAG | 29 |
| IDT8 | Y | chr6 | CATACAGTGCCCCCTCCCTAAG | AGCTTTCCTGAGGGGCATTCTC | 33 |
| D1Mit426 | R | chr1 | CTGCCATCCACTACTTGGTG | CAAATGATACAGTGGGAAACCCC | 15 |
| M16 | R | chr10 | TTCAGGTAGATACATCAGACCTGTGG | AAGTCTTGGGGGAACAGTCGAG | 22 |
| M13 | R | chr14 | GGGCATAAATTGTTTGTCGCTTG | GTGTGACTGCTCGCTTCCCATC | 31 |
| L2140 | R | chr19 | GAGAAACCCTGTCTCGAAAAGAAG | GTCGACGCTGCAACTCAGTC | 42 |
